# Normal X-inactivation mosaicism in corneas of heterozygous *Flna^Dilp2/+ ^*female mice--a model of human Filamin A (*FLNA*) diseases

**DOI:** 10.1186/1756-0500-5-122

**Published:** 2012-02-27

**Authors:** Panagiotis Douvaras, Weijia Liu, Richard L Mort, Lisa McKie, Katrine M West, Sally H Cross, Steven D Morley, John D West

**Affiliations:** 1 Division of Reproductive and Developmental Sciences, Genes and Development Group, University of Edinburgh, Hugh Robson Building, George Square, Edinburgh EH8 9XD, UK; 2 Centre for Integrative Physiology, University of Edinburgh, Hugh Robson Building, George Square, Edinburgh EH8 9XD, UK; 3 Medical and Developmental Genetics Section, MRC Human Genetics Unit, MRC IGMM, University of Edinburgh, Western General Hospital, Crewe Road, Edinburgh EH4 2XU, UK; 4 Clinical Biochemistry Section, School of Clinical Sciences and Community Health, Queen's Medical Research Institute, University of Edinburgh, 47 Little France Crescent, Edinburgh EH16 4TJ, UK; 5 The New York Stem Cell Foundation, 3960 Broadway, 4th Floor, Suite 440B, New York, NY 10032, USA

## Abstract

**Background:**

Some abnormalities of mouse corneal epithelial maintenance can be identified by the atypical mosaic patterns they produce in X-chromosome inactivation mosaics and chimeras. Human *FLNA*/+ females, heterozygous for X-linked, filamin A gene (*FLNA*) mutations, display a range of disorders and X-inactivation mosaicism is sometimes quantitatively unbalanced. *Flna*^*Dilp2/+ *^mice, heterozygous for an X-linked filamin A (*Flna*) nonsense mutation have variable eye, skeletal and other abnormalities, but X-inactivation mosaicism has not been investigated. The aim of this study was to determine whether X-inactivation mosaicism in the corneal epithelia of *Flna*^*Dilp2/+ *^mice was affected in any way that might predict abnormal corneal epithelial maintenance.

**Results:**

X-chromosome inactivation mosaicism was studied in the corneal epithelium and a control tissue (liver) of *Flna*^*Dilp2/+ *^and wild-type (WT) female X-inactivation mosaics, hemizygous for the X-linked, *LacZ *reporter H253 transgene, using β-galactosidase histochemical staining. The corneal epithelia of *Flna*^*Dilp2/+ *^and WT X-inactivation mosaics showed similar radial, striped patterns, implying epithelial cell movement was not disrupted in *Flna*^*Dilp2/+ *^corneas. Corrected stripe numbers declined with age overall (but not significantly for either genotype individually), consistent with previous reports suggesting an age-related reduction in stem cell function. Corrected stripe numbers were not reduced in *Flna*^*Dilp2/+ *^compared with WT X-inactivation mosaics and mosaicism was not significantly more unbalanced in the corneal epithelia or livers of *Flna*^*Dilp2/+ *^than wild-type *Flna^+/+ ^*X-inactivation mosaics.

**Conclusions:**

Mosaic analysis identified no major effect of the mouse *Flna^Dilp2 ^*mutation on corneal epithelial maintenance or the balance of X-inactivation mosaicism in the corneal epithelium or liver.

## Background

Filamin A is a cytoskeleton protein with multiple roles that binds to actin and other proteins in many cell types. It is encoded by an X-linked gene, designated *FLNA *in humans and *Flna *in mice; mutations in human *FLNA *cause several types of congenital birth defects [1,2]. Females heterozygous for a loss of function mutation are viable but have periventricular nodular heterotopia (PVNH), a developmental defect in neuronal migration, whereas hemizygous males do not survive. *FLNA *missense mutations cause four genetic syndromes belonging to the otopalatodigital (OPD) spectrum disorders. The phenotypes of heterozygous females vary according to the syndrome but include skeletal dysplasia, urogenital defects and deafness. Hemizygous males with the more severe syndromes die *in utero *or shortly after birth but those with the mildest syndrome survive.

A mutant *Flna *mouse with misshapen pupils was recovered from a mutagenesis screen [3]. This new mutant was initially called *Dilp2 *(dilated pupils 2) but renamed *Flna^Dilp2 ^*once it was mapped to the *Flna *locus and the underlying defect identified [4]. The *Flna^Dilp2 ^*mutation results in nonsense-mediated decay of the *Flna *mRNA and, therefore, absence of any Flna protein. Heterozygous *Flna*^*Dilp2/+ *^female mice are mostly viable and fertile but have mild defects of the eyes, sternum and palate whereas *Flna^Dilp2^*/Y males die *in utero *with heart defects [4]. After X-chromosome inactivation, heterozygous *Flna*^*Dilp2/+ *^and *FLNA/+ *females are X-inactivation mosaics with two genetically distinct cell populations. Although the *Flna*^*Dilp2/+ *^phenotype has been characterised [4], X-inactivation mosaicism was not investigated. This has been reported to be very unbalanced in some human *FLNA/+ *females [1,5]. Unbalanced X-inactivation mosaicism may be caused by primary non-random X-chromosome inactivation (e.g. heterozygosity for different *Xce *alleles in mice [6-11]) or by various secondary selection processes.

Some abnormalities of mouse corneal epithelial maintenance can be identified by the abnormal mosaic patterns they produce in X-chromosome inactivation mosaics and chimeras. According to the widely accepted, limbal epithelial stem cell (LESC) hypothesis, the corneal epithelium is maintained by stem cells located in the limbus between the cornea and conjunctiva [12,13]. These produce daughter transient (or transit) amplifying cells (TACs), which move centripetally in the basal epithelial layer and divide several times before they leave the basal layer and move apically towards the surface from where they are shed. Mosaic patterns in the adult corneal epithelia of wild-type (WT), adult mouse chimeras, X-inactivation mosaics and other types of mosaics change from a randomly orientated patchwork to radial stripes between 5 and 8 weeks after birth [14-17]. This change in pattern is thought to reflect the activation of LESCs at the corneal periphery and the radial stripes are presumed to be formed by lineages of TACs, which move centripetally from the limbus towards the centre of the cornea. This is consistent with more direct evidence for centripetal movement of corneal epithelial cells [18,19]. The radial striped pattern is disrupted in some mutant mice, including *Pax6^+/Sey-Neu ^*and *Pax6^+/Leca4 ^*X-inactivation mosaics [20,21] and transgenic mosaics that are also *Dstn*^*corn1/corn1 *^homozygotes [22], implying that cell movement is affected. Numerical analysis of these striping patterns provides an indirect estimate of the number of coherent clones of LESCs maintaining the corneal epithelium [14,16] and has been used to predict that stem cell function declines with age and may already be reduced in young (15-week) *Pax6^+/Sey-Neu ^*mice [20,21].

Although the radial striped pattern is disturbed in *Pax6^+/Sey-Neu ^*X-inactivation mosaics, where all the cells are *Pax6^+/Sey-Neu^*, it is normal when *Pax6^+/Sey-Neu ^*and WT cells are combined in *Pax6^+/Sey-Neu ^*↔ WT chimeras. This suggests that the presence of WT cells can rescue the defect in cell movement [20]. However, the *Pax6^+/Sey-Neu ^*cells were under-represented in corneal epithelia, relative to other tissues in these chimeras, implying that active *Pax6^+/Sey-Neu ^*LESCs were depleted.

X-chromosome linkage of the *Flna^Dilp2 ^*mutation provides an opportunity to use X-chromosome inactivation mosaics to investigate the effect of the mutant *Flna^Dilp2 ^*allele on stem cell function in ways that would normally require production of experimental chimeras. *Flna^Dilp2 ^XLacZ^-^*/*Flna^+ ^XLacZ^Tg ^*X-inactivation mosaics are expected to be equivalent to chimeras with separate populations of β-galactosidase (β-gal)-positive cells expressing the WT, *Flna^+ ^*allele and β-gal-negative cells expressing the mutant *Flna^Dilp2 ^*allele. If expression of the *Flna^Dilp2 ^*mutation affected stem cell function, β-gal-negative cells (expressing *Flna^Dilp2^*) would be expected to be under-represented in corneal epithelia, relative to other tissues in *Flna^Dilp2 ^XLacZ^-^*/*Flna^+ ^XLacZ^Tg ^*X-inactivation mosaics in the same way that *Pax6^+/Sey-Neu ^*cells were under-represented in corneal epithelia, relative to other tissues in *Pax6^+/Sey-Neu ^*↔ WT chimeras [20].

The aim of the present study was to identify whether X-inactivation mosaicism in the corneal epithelia of *Flna*^*Dilp2/+ *^heterozygotes was abnormal in any way that predicted that *Flna *has a role in LESC function or corneal maintenance.

## Methods

### Mice and genotyping

Animal work was performed in accordance with institutional guidelines and UK Home Office regulations. *Flna*^*Dilp2/+ *^mice were maintained by crossing *Flna*^*Dilp2/+ *^females with WT C57BL/6 or (C57BL/6 × CBA/Ca)F1 males. Females for analysis were produced by crossing *Flna*^*Dilp2/+ *^females with *XLacZ^Tg^*/Y, H253 strain males [23,24], which ubiquitously express an X-linked nLacZ transgene (abbreviated to *XLacZ^Tg ^*to produce *Flna^Dilp2 ^XLacZ^- ^/Flna^+ ^XLacZ^Tg ^*and wild-type *Flna^+ ^XLacZ^- ^/Flna^+ ^XLacZ^Tg ^*females. Both groups of females were X-inactivation mosaics and showed mosaic transgene expression of the *XLacZ^Tg ^*transgene, after X-gal staining for β-galactosidase (β-gal) activity.

*Flna^Dilp2/+ ^*and *Flna^+/+ ^*females were distinguished by PCR of *Flna *exon 44 DNA from ear clip biopsies. DNA was prepared using direct PCR lysis reagent (Viagen 401-E). 1 μl DNA was used in 20 μl PCR reaction mixtures containing forward primer TGA AGG GGA TGT TAA CCA ATT C, reverse primer TCT ATC TCA CTG GCT TCC TTG C and DNA polymerase (BIO-X-ACT, Bioline). DNA was amplified in a thermocycler by incubating at 94°C for 3 min., followed by 30 cycles of 30 s at 94°C, 45 s at 55°C, 1 min. extension at 68°C and a final incubation of 2 min at 68°C. The Dilp2 mutation creates an AluI restriction site. Therefore, the PCR products were digested with AluI restriction enzyme at 37°C for 2 h., separated by electrophoresis on 2% agarose gels, containing ethidium bromide, and visualised on a UV transilluminator.

### β-galactosidase staining and analysis of X-inactivation mosaicism

For analysis of corneas, mice were culled and the eyes were fixed in 0.2% glutaraldehyde solution, stained overnight in X-gal staining solution as described previously [14], post-fixed in 4% paraformaldehyde and stored in 70% ethanol. Mosaic patterns in the adult corneal epithelium occurred as radial stripes and were analysed as described previously to estimate the percentage of β-gal-positive cells and the 'corrected stripe number' [14,16,20,21] except that a semi-automated method was used for analysis [25]. This involved using the 'Circular Clonal Analysis' tool provided by the ClonalTools ImageJ plugin (http://imagejdocu.tudor.lu/doku.php?id=plugin:analysis:clonaltools:start) to define a circular line selection that was concentric with the corneal epithelium. To avoid errors arising from sampling too close to the limbal epithelium, the radius of the circle was only 80% of the corneal radius. Lengths of β-gal-positive and β-gal-negative tissue were measured along the circular line selection (across the widths of the stripes) to estimate the percentage of β-gal-positive cells and the observed mean stripe width [21,25].

The corrected stripe number adjusts for the likely mean number of adjacent radial, β-gal-positive corneal epithelial clones within a single β-gal-positive radial stripe. The corrected mean stripe width is derived by dividing the observed mean stripe width by the function 1/(1-*p*), where *p *is the proportion of β-gal-positive cells around the circumference, as described previously [14,16]. If the corrected mean stripe width is expressed as a percentage of the circumference of the corneal epithelium, it can be readily converted to the corrected stripe number per circumference. This provides an estimate of the total number of active corneal epithelial coherent clones (both β-gal positive and β-gal negative) per circumference. The corrected stripe number is useful for comparing numbers of active clones of stem cells between different groups because it is believed that each coherent clone in the corneal epithelium is maintained by a coherent clone of stem cells in the limbus at the periphery of the cornea. However, because the number of stem cells per coherent clone may vary it is not a direct estimate of the number of active stem cells [14,16].

Livers were analysed as frozen sections. Mice were culled and their livers were removed, washed with cold PBS, cut into small pieces and fixed in 4% paraformaldehyde (2 h. at 4°C). Fixed samples were washed in PBS (3 × 10 min), transferred to 30% glycerol in PBS (4°C, overnight), 50% glycerol (4°C, overnight) and stored in 80% glycerol at 4°C. Before sectioning, tissues were re-hydrated (50% and 30% glycerol for 1 h. each and then to PBS for 30 min.). Samples were immersed in OCT embedding medium in plastic moulds and snap-frozen on dry ice. Frozen sections were cut at 10 μm thickness, transferred to Polysine slides and stained for β-gal activity using a modification of an established protocol [14]. Slides were soaked in wash buffer (0.1 M Na phosphate, 2 mM MgCl_2_, 0.01% sodium deoxycholic acid and 2% Igepal) 3 × 10 min., then incubated in X-gal staining solution at 37°C in the dark overnight. After staining, slides were washed in PBS (3 × 10 min) and counterstained with 1% neutral red in 1.48 mM acetate buffer, pH 4.8 for 5 min. and rinsed in water. Sections were dehydrated rapidly through a graded ethanol series, cleared in xylene and mounted with DPX mounting medium (BDH, Poole, UK) and cover slips. β-gal positive and β-gal negative cells were counted in five square scoring regions in one section from each sample (Zeiss Axioplan-2 microscope; ×40 objective). Random number tables were used to position each scoring region randomly on the section. Each scoring region, containing ~200 cells, was divided into 100 smaller squares, with a 10 × 10 eyepiece graticule, for cell counting and the person scoring was blinded to the genotypes.

### Statistical analysis

Genotype frequencies were compared to Mendelian expectations by goodness of fit *χ*^2 ^tests. Percentages of β-gal positive cells and corrected stripe numbers in different groups were compared by 2-way analysis of variance (ANOVA) using GraphPad Prism software. For β-gal positive cells and corrected stripe numbers in the corneal epithelium, individual values analysed were means of left and right eyes of each mouse.

## Results

### Survival of *Flna*^*Dilp2*/+ ^heterozygotes

Crosses between *Flna*^*Dilp2*/+ ^females with *XLacZ^Tg^*/Y males produced 50 *Flna^Dilp2/+ ^*females, 88 wild-type *Flna^+/+ ^*females and 81 males (not genotyped) at weaning. The frequencies of the two female genotypes differed significantly from a 1:1 ratio (*χ*^2 ^= 10.46; *P *= 0.0012) and implied a 43% deficiency of *Flna*^*Dilp2*/+ ^females, which is consistent with previously reported findings [4]. It was shown previously that *Flna^Dilp2^*/Y males die before birth [4]. The frequencies of surviving males and *Flna^+/+ ^*females differed significantly from the 2:1 ratio expected if all males survived (*χ*^2 ^= 26.70; *P *< 0.0001) but did not differ significantly from a 1:1 ratio (*χ*^2 ^= 0.290; *P *= 0.590), which is consistent with the previously reported lethality of *Flna^Dilp2^*/Y males [4].

### X-inactivation mosaicism in the corneal epithelium

We compared X-chromosome mosaicism in corneal epithelia and a control tissue (liver) from *Flna^Dilp2 ^XLacZ^- ^/Flna^+ ^XLacZ^Tg ^*and *Flna^+ ^XLacZ^- ^/Flna^+ ^XLacZ^Tg ^*X-inactivation mosaic females, as described in the Methods. Cells expressing the X-chromosome carrying the *XLacZ^Tg ^*transgene were identified as β-gal-positive by histochemistry [23].

**Figure 1:**
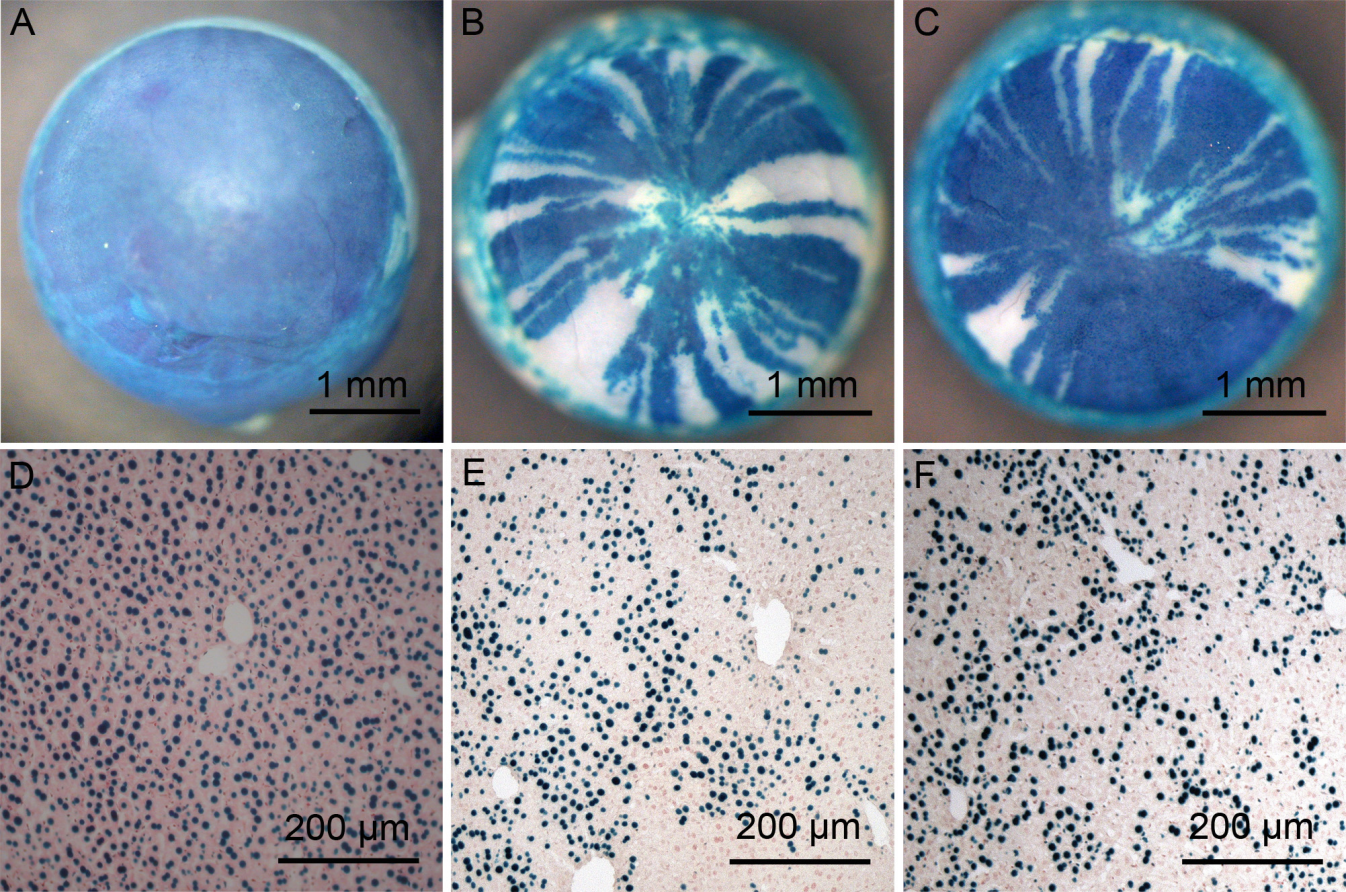
**β-gal staining of tissues from *Flna*^*Dilp2*/+ ^and wild-type *Flna^+/+ ^*X-inactivation mosaics**. (**A-C**) Corneas from (**A**) an *XLacZ^Tg/Tg ^*positive control (100% β-gal-positive), (**B**) a 15-week, WT (*Flna^+/+^*) *XLacZ^Tg/- ^*X-inactivation mosaic (68.7% β-gal-positive) and (**C**) a 15-week, *Flna^Dilp2/+ ^*X-inactivation mosaic (84.5% β-gal-positive). (B) and (C) show similar radial stripes of β-gal-positive (blue) and β-gal-negative (unstained) cells. (**D-F**) Sections of livers from (**D**) *XLacZ^Tg/Tg ^*positive control (100% β-gal-positive), (**E**) a WT X-inactivation mosaic and (**F**) a *Flna^Dilp2/+ ^*X-inactivation mosaic. (E) and (F) show similar randomly orientated patches of β-gal-positive (blue) and β-gal-negative (pink) cells.

Non-mosaic *XLacZ^Tg^*/Y males and *XLacZ*^*Tg*/*Tg *^females showed uniform blue staining in the cornea (Figure 1A). When the intact eye is stained all 4-6 layers of the corneal epithelium become stained but the underlying stroma remains unstained unless the epithelium is damaged [14,16]. Mosaic patterns in the corneal epithelium were examined in 15- and 30-week old adults, by which time radial stripe patterns are established in WT corneas [14,16,20,21]. Both heterozygous *Flna*^*Dilp2*/+ ^and WT X-inactivation mosaics showed normal radial striped patterns (Figure 1B, C).

**Figure 2:**
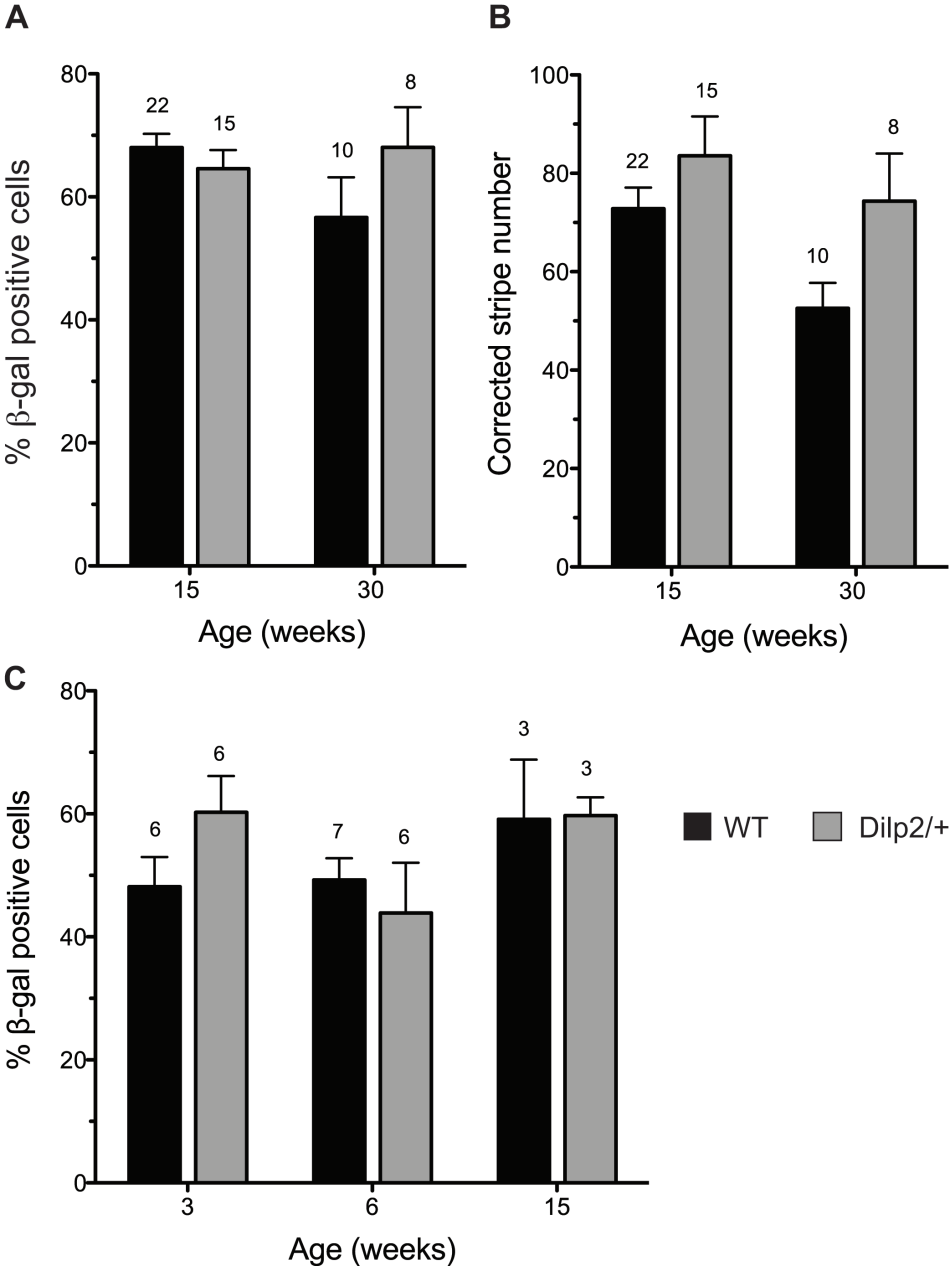
**Quantitative comparison of heterozygous *Flna*^*Dilp2*/+ ^and wild-type *Flna^+/+ ^*X-inactivation mosaics**. (**A**) Percentage of β-gal-positive cells in corneal epithelia from *Flna^Dilp2 ^*and WT (*Flna^+/+^*) X-inactivation mosaics at 15 and 30 weeks. (Individual values were means of left and right eyes of each mouse.) There were no significant differences between genotypes or ages by 2-way ANOVA. (**B**) Corrected stripe numbers in corneal epithelia from *Flna*^*Dilp2*/+ ^and WT X-inactivation mosaics at 15 and 30 weeks. (Individual values were means of left and right eyes of each mouse.) Statistical analysis showed significant differences between both ages (*P *= 0.040) and genotypes (*P *= 0.024) by 2-way ANOVA. However, neither age nor genotype differences were significant for individual pairwise comparisons when analysed separately by Bonferroni post-hoc tests. (**C**) Percentage of β-gal-positive cells in livers from *Flna*^*Dilp2*/+ ^and WT X-inactivation mosaics at 3, 6 and 15 weeks. There were no significant differences between genotypes or ages by 2-way ANOVA. In each case the results are presented as mean ± SEM and the number of mice is shown above each bar.

Quantitative analysis was based on means of left and right eyes for each mouse with 8-22 mice per group as shown in Figure 2. A 2-way ANOVA showed there were no overall significant differences, in percentage of β-gal-positive cells (expressing the WT, *Flna^+ ^*allele) in the corneal epithelium (Figure 2A), for age (*P *= 0.347) or genotype (*P *= 0.342) and the interaction was also non-significant (*P *= 0.081). Thus, the percentage of β-gal-positive cells was not significantly greater in *Flna^Dilp2/+ ^*than WT X-inactivation mosaics at either 15 or 30 weeks. Overall, a 2-way ANOVA showed that the 'corrected stripe number' (Figure 2B) declined significantly with age (*P *= 0.040), as previously reported for WT X-inactivation mosaics [16], and was significantly higher in *Flna^Dilp2/+ ^*than WT X-inactivation mosaics (*P *= 0.024) but the interaction was non-significant (*P *= 0.434). However, age differences were not significant for either genotype separately and genotype differences were not significant when 15- and 30-week old mice were analysed separately by Bonferroni post-hoc tests.

### X-inactivation mosaicism in the liver

More than 98% of cells in frozen sections of livers from non-mosaic *XLacZ^Tg^*/Y males and *XLacZ^Tg/Tg ^*females were β-gal-positive so we used the liver as a control tissue and quantitatively compared the mosaicism in livers of *Flna*^*Dilp2*/+ ^and *Flna*^*+*/+ ^female, X-inactivation mosaics (Figure 1D). Staining patterns in mosaic livers were examined at three, six and 15 weeks (Figure 1E, F) in an attempt to identify whether any unbalanced mosaicism resulted from cell selection over this period. A 2-way ANOVA on the results of a quantitative analysis (Figure 2C) revealed no significant differences in the percentage of β-gal-positive cells in livers of *Flna*^*Dilp2*/+ ^and *Flna*^*+*/+ ^X-inactivation mosaics at any of the ages examined. For age comparisons *P *= 0.161, for genotypes *P *= 0.652 and for the interaction, *P *= 0.307.

## Discussion

Our study revealed no significant adverse effect of the *Flna*^*Dilp2*/+ ^genotype on X-inactivation mosaicism in either the corneal epithelium or liver. *Flna*^*Dilp2*/+ ^heterozygotes had qualitatively normal radial striped patterns, implying that corneal epithelial cell movement was normal. Although, overall the corrected stripe numbers were higher in *Flna*^*Dilp2*/+ ^corneas than WT, they were within the WT range observed previously [14,16,20,21]. As *Flna*^*Dilp2*/+ ^corrected stripe numbers were not reduced, there was no evidence from the mosaic analysis for any overall reduction in stem cell function in *Flna*^*Dilp2*/+ ^corneas. Furthermore, as β-gal-negative cells, expressing the *Flna^Dilp2 ^*mutation were not significantly under-represented in the corneal epithelium, there was no evidence that stem cells expressing the *Flna^Dilp2 ^*mutation were depleted or less active than those expressing the WT allele. We conclude that mosaic analysis identified no major effect of the mouse *Flna^Dilp2 ^*mutation on corneal epithelial cell movement or maintenance of the corneal epithelium by clones of limbal epithelial stem cells.

A previous investigation estimated lethality among heterozygous *Flna*^*Dilp2*/+ ^females as approximately 30% [4] and in the present study we estimated this loss as about 43%. Hart *et al*. suggested that survival might be lower for *Flna*^*Dilp2*/+ ^females with a higher proportion of cells with the mutant X-chromosome active [4]. This predicts that the average percentage of β-gal-positive cells (not expressing the *Flna^Dilp2 ^*mutation) would be higher among surviving heterozygous *Flna*^*Dilp2*/+ ^mosaic females than among the WT *Flna*^*+*/+ ^mosaic females, either generally or in one or more critical tissue(s). This was not the case for either the corneal epithelium or liver, so there is no evidence that the composition of these tissues correlates with anything that affects the survival of *Flna*^*Dilp2*/+ ^mosaic females.

The failure to detect an imbalance in X-inactivation mosaicism in the *Flna*^*Dilp2*/+ ^mouse liver or corneal epithelium, as reported for leukocytes from human *FLNA/+ *females [1,5], could reflect the type of mutation, species differences and/or the tissues studied. Although heterozygotes for *FLNA *mutations causing OPD spectrum disorders showed a pronounced imbalance in X-inactivation mosaicism in leukocytes [1,5] this does not appear to be true of heterozygotes for mutations causing PVNH [26]. *Flna^Dilp ^*is a loss of function mutation and, therefore, is more similar to *FLNA *mutations causing PVNH than those causing OPD disorders but the mouse *Flna*^*Dilp*/+ ^phenotype differs from the human PVNH phenotype [4]. It may be informative to compare the phenotypes of other mouse *Flna *alleles and their effects on X-inactivation mosaicism and to extend the range of tissues examined.

## Conclusions

Analysis of patterns of X-inactivation mosaicism revealed no evidence that mouse *Flna^Dilp2 ^*mutation has a major detrimental effect on maintenance of the corneal epithelium by clones of limbal epithelial stem cells. In contrast to some reports for human *FLNA/+ *heterozygotes, there was no evidence for unbalanced X-inactivation mosaicism in the tissues we examined from *Flna^Dilp2/+ ^*mice.

## Abbreviations

ANOVA: Analysis of variance; ß-gal: ß-galactosidase; LESC: Limbal epithelial stem cell; OPD: Otopalatodigital; PBS: Phosphate buffered saline; PVNH: Periventricular nodular heterotopia; SEM: Standard error of the mean; TAC: Transient (or transit) amplifying cell; UV: Ultraviolet; WT: Wild-type; X-gal: Bromo-chloro-indolyl-galactopyranoside; *XLacZ^Tg/-^*: Female X-inactivation mosaic mice: hemizygous for the H253 X-linked *nLacZ *transgene.

## Competing interests

The authors declare that they have no competing interests.

## Authors' contributions

PD carried out the staining and photography of the mosaic corneas, established the protocol for staining frozen sections, stained some of the tissue sections and contributed to the data analysis and the draft manuscript, WL carried out staining and analysis of the livers, RM carried out the quantitative analysis of the corneal epithelia, LM and KMW genotyped the mice, SHC managed the primary *Flna^Dilp2 ^*mouse colony, SDM participated in the study design and helped to draft the manuscript. JDW conceived and coordinated the study and wrote most of the first draft of the manuscript. All authors read and approved the final manuscript.
